# Novel Biological Approaches for Testing the Contributions of Single DSBs and DSB Clusters to the Biological Effects of High LET Radiation

**DOI:** 10.3389/fonc.2016.00163

**Published:** 2016-06-28

**Authors:** Veronika Mladenova, Emil Mladenov, George Iliakis

**Affiliations:** ^1^Institute of Medical Radiation Biology, University of Duisburg-Essen Medical School, Essen, Germany

**Keywords:** radiation effects, high-LET, RBE, DSB clusters, DSB repair, I-*Sce*I, CRISPR/Cas9

## Abstract

The adverse biological effects of ionizing radiation (IR) are commonly attributed to the generation of DNA double-strand breaks (DSBs). IR-induced DSBs are generated by clusters of ionizations, bear damaged terminal nucleotides, and frequently comprise base damages and single-strand breaks in the vicinity generating a unique DNA damage-clustering effect that increases DSB “complexity.” The number of ionizations in clusters of different radiation modalities increases with increasing linear energy transfer (LET), and is thought to determine the long-known LET-dependence of the relative biological effectiveness (RBE). Multiple ionizations may also lead to the formation of DSB clusters, comprising two or more DSBs that destabilize chromatin further and compromise overall processing. DSB complexity and DSB-cluster formation are increasingly considered in the development of mathematical models of radiation action, which are then “tested” by fitting available experimental data. Despite a plethora of such mathematical models the ultimate goal, i.e., the *“a priori”* prediction of the radiation effect, has not yet been achieved. The difficulty partly arises from unsurmountable difficulties in testing the fundamental assumptions of such mathematical models in defined biological model systems capable of providing conclusive answers. Recently, revolutionary advances in methods allowing the generation of enzymatic DSBs at random or in well-defined locations in the genome, generate unique testing opportunities for several key assumptions frequently fed into mathematical modeling – including the role of DSB clusters in the overall effect. Here, we review the problematic of DSB-cluster formation in radiation action and present novel biological technologies that promise to revolutionize the way we address the biological consequences of such lesions. We describe new ways of exploiting the I-*Sce*I endonuclease to generate DSB-clusters at random locations in the genome and describe the possible utility of Zn-finger nucleases and of TALENs in generating DSBs at defined genomic locations. Finally, we describe ways to harness the revolution of CRISPR/Cas9 technology to advance our understanding of the biological effects of DSBs. Collectively, these approaches promise to improve the focus of mathematical modeling of radiation action by providing testing opportunities for key assumptions on the underlying biology. They are also likely to further strengthen interactions between experimental radiation biologists and mathematical modelers.

## Introduction

All living organisms are continuously exposed to background ionizing radiation (IR) deriving from space, solar activity, or emitted by certain minerals and soils. Although it is generally accepted that IR “*per se*” can be harmful, IR is nevertheless extensively used for diagnostic purposes and in cancer therapy. Therefore, it is of a great importance to investigate and rationalize the mechanisms of IR action on living organisms, as this will directly help to maximize human radiation protection and to optimize approaches to cancer treatment.

Ionizing radiation generates a broad spectrum of DNA damages, encompassing single-strand breaks (SSB), a variety of oxidative base lesions, DNA–DNA crosslinks as well as DNA–Protein crosslinks, and double-strand breaks (DSBs) (1, 2). However, from the variety of lesions induced by IR, the DSB elicits most of the documented detrimental effects (3, 4), including genomic rearrangements, chromosome aberrations, cell death, genetic mutations, and cancer (5–8).

It has long been known that different IR modalities generate markedly different biological responses although they generate in principle the same basic lesions described above. Thus, α-particles, neutrons, or high-charge and energy particles (HZE ions) are significantly more effective in killing cells than high-energy electrons or protons, γ-rays, or X-rays (9, 10). This increased efficacy, typically described by the higher relative biological effectiveness (RBE), depends on the linear energy transfer (LET) of the radiation modality – which for charged particles is the energy absorbed per unit particle track-length, expressed as kiloelectron-volts/micrometer. Typically, RBE increases with increasing LET of radiation up to a maximum and declines subsequently (11–14).

In the recent years, charged particles such as carbon ions are increasingly considered as main modality for cancer radiotherapy and inflammation treatment in an effort to harness in a targeted manner their higher LET (15–18).

## Complexity of IR Induced DSBs: The Role of LET

Bacteria harness the severity of DSB as a lesion to protect themselves from foreign DNA. A family of enzymes, known as restriction endonucleases (RE), recognize and cut specific DNA sequences generating DSBs with blunt or staggered ends. During this process, nucleotides are not altered and the phosphodiester bond retains the 5′-phosphate and 3′-OH groups at each DNA strand. As a result, processing and removal of the DSB by simple ligation is in principle possible. RE-generated DSBs have been used to model IR-induced DSBs (see below) (19, 20) and found to have reparability that depends on the type of ends generated (21–23).

The approach to model DSBs using nucleases gained ground with the introduction of I-*Sce*I homing endonuclease, whose 18-bp long recognition sequence (5′-TAGGGATAA/CAGGGTAAT-3′) is not present in the mammalian genome. The I-*Sce*I recognition sequence can nevertheless be inserted into a mammalian genome according to a pre-conceived design using molecular biology approaches (24–27). I-*Sce*I recognition sequences artificially introduced in a genome can be cut to generate DSBs by expressing constitutive or inducible forms of the endonuclease (28, 29). The biological consequences of these DSBs can then be analyzed using molecular biology approaches. The strength of the method lies in the fact that DSBs are generated at defined locations in the genome, and that combination with appropriately constructed reporters allows analysis of the underlying processing mechanisms.

When DSBs are induced by IR *via* oxidation reactions – either direct loss of an electron or attack from an ^⋅^OH produced from the radiolysis of water – they frequently comprise ends with a 3′-damaged sugar in the form of phosphoglycolate and a 5′-OH groups (30–33). Such ends prevent direct DNA ligation and necessitate end-processing during repair (34). Moreover, the adverse biological effects of X-rays or γ-rays are thought to derive from DSBs generated within ionization clusters (35, 36), and not by the coincidence of independently generated ionizations on opposite DNA strands. Indeed, track-structure calculations using computational approaches (37–39) show that secondary electrons, at the end of their tracks generate clusters of ionizations, i.e., multiple ionizations confined in a small volume.

Despite the generation of ionization clusters at the ends of low-energy electron tracks, X-rays and γ-rays still deposit 50–70% of their energy in well-separated ionization events that generate a relatively even ionization pattern within the cell (35, 36). Consequently, X-rays and γ-rays are considered low-LET forms of IR. On the other hand, charged particles (e.g., α-particles or carbon ions) are considered as high-LET forms of radiation because they ionize along their tracks at a higher rate than the electrons generated by X-rays (40).

This increased clustering of ionizations generates DNA damage that is more complex than that induced by low-LET radiations, in the sense that it comprises more DNA lesions within one or two turns of the DNA helix (33). It constitutes what is sometimes called clustered damage sites (CDS) or multiply damaged sites (MDS) (41, 42). While MDS are generated by low-LET radiation such as X-rays, they occur more frequently after exposure to high-LET radiations and are implicated in their enhanced biological effects.

Indeed, about 30% of DSBs contain additional lesions following exposure to low-energy electrons; notably, this fraction increases up to 70% at the same dose of α-particles. In addition, the ratio of the number of SSBs to DSBs decreases from 22.8 for ^60^Co γ-rays to 3.4 for 50 MeV ^12^C-ions (39, 43). Since these shifts in the spectrum of lesions do not increase the yields of DSBs in a manner corresponding to the increased killing after exposure to high- versus low-LET radiation, it can be inferred that increased clustering of DNA damage is an important determinant of the biological effect (see also below) (44).

Complexity at a DSB may compromise repair through the simultaneous recruitment of multiple repair-pathway-factors (e.g., from one of the DSB repair pathways together with factors of BER) to close-by lesions in the DNA. Moreover, it may even generate a DSB indirectly when in a complexly damaged DNA, individual lesions in the two strands are processed independently (6, 43, 45, 46). There is evidence that this form of clustered DNA damage outnumbers direct DSBs after exposure to low-LET radiation by nearly 4:1. Similarly, delayed formation of DSBs can occur from the chemical evolution in cells of thermally unstable lesions, which initially do not break the DNA, but which do so minutes after irradiation as they become chemically modified in the cellular milieu (47–52). DSB repair models considering DSB complexity have been also developed to describe radiation effects and DSB repair kinetics throughout the cell cycle (53).

It is thus evident that IR-induced DSBs are the products of ionization clusters that generate clustered DNA damage, which can present in different forms of complexity including modified ends, presence of other lesions in the vicinity of the break, as well lesions that generate DSBs only after enzymatic or chemical processing. Since the size of ionization clusters that generate complex DSBs increases with increasing LET, it is plausible to consider this form of DNA damage complexity as a relevant determinant of the increased effectiveness of high-LET radiation.

## Higher Order of DNA Damage Complexity: DSB Clusters

An additional level of DSB complexity is generated by clusters of DSBs (33, 54). This form of DNA damage severely undermines local chromatin stability and thus overall processing in a chromatin-location and composition-dependent manner. DSB clustering as a cause of irreversible radiation effects has been considered by several investigators [see Ref. (39) for a review]. Thus, Bryant and his group developed a non-ionic neutral filter elution assay to generate histone-depleted nuclear structures retaining higher order nuclear matrix organization, and used it to measure DNA fragment loss from two or more DSBs within a single-looped chromatin domain (55–57). They proposed that the spatial distribution of DSBs in higher order chromatin loops affects their reparability, and that misrepair involves DNA fragment-loss at such DSB clusters.

Holley and Chatterjee also considered DSB clusters as a particularly consequential form of radiation damage particularly for high-LET radiations (58). In their calculations, they found fragmentation peaks at 85 bp and then again at multiples of 1000 bp, which they interpreted as reflecting aspects of chromatin structure. Notably, such fragments could indeed be detected by pulsed-field gel electrophoresis in irradiated cells (59, 60) and have also been postulated using alternative modeling approaches (14, 39, 61, 62).

Atomic force microscopy imaging shows clustered DSBs and formation of short DNA fragments – even when irradiating “naked” DNA (63). Small (<30 bp) DNA fragments generated from clustered DSBs have also been propose to compromise Ku function (64). Further work shows that DNA–PK, a complex between the Ku and DNA–PKcs, is also inhibited by short (14–20 bp) DNA fragments (63).

The contribution of DSB clusters to the adverse effects of IR has been the focus of extensive mathematical modeling (39). Ostashevsky developed a model according to which DSB clusters generate small DNA fragments, which can be lost from the chromatin context, thus compromising repair of the constituent DSBs (65, 66). A more specialized induction of DSB clusters within chromatin loops, similar to that considered by Bryant, has been used to develop alternative mathematical models (39, 61, 62, 67, 68). In addition, Scholz and his group (69–71) use an extension of the Giant LOop Binary LEsion (GLOBLE) model (72) and classify DNA lesions with respect to their distribution in giant chromatin loops as single DSBs or DSB clusters (~2 Mbp in size) (73–75). These assumptions generally allow successful fitting of cell survival data (76, 77), including fluctuations of radiosensitivity throughout the cell cycle (72).

Mathematical models to analyze DSB repair kinetics based on DSB complexity have been also recently developed (78). In the synapsis formation (SF) model, the rejoining of complex DSBs is not simulated as a first order event (break filling/joining). Rather the rejoining of complex DSBs is assumed to be realized through SF, similar to a second-order reaction between DNA ends. This approach allows DNA ends to be clearly defined before the SF, which is essential for predicting higher number of chromosomal aberrations after high- as compared to low-LET radiation.

Notably, the generation of DSB clusters represents a form of chromothripsis, defined as chromosome shattering and subsequent incorrect rejoining that underpins carcinogenesis (79–82).

The satisfactory fitting of experimental data achieved under these assumptions points to the biological relevance of DSB clusters as a level of DNA damage complexity that likely explains the increased biological efficacy of high LET radiation.

## Mathematical Modeling of Radiation Action Will Benefit from Molecular Biology Approaches Directly Testing Their Basic Assumptions

Collectively, the above outline shows how the physical clustering of ionizations generates DSBs of different complexity, as well as DSB-clusters, and places these forms of DNA damage to the center of responses elicited by radiation modalities of different LET. The recognition that discontinuities in the genome may be caused by DSBs of widely different complexity, immediately implies different biological consequences.

Information on the molecular underpinnings of the responses elicited by genomic breaks of different complexity is scarce despite the central contribution widely attributed to this parameter in the overall radiation effect. As a result, DNA damage complexity is typically only mathematically “modeled” in radiation response formalisms, without direct knowledge of the biological effects of each complexity level. As a consequence, quality of fitting is the only way for testing the validity of the basic assumptions on which these models rest. Yet, this approach is not satisfactory due to the large spectrum of DNA damages induced by IR and their dependence on LET that increases the number of parameters required for complete mathematical modeling.

Furthermore, IR-dependent DSB induction by nature precludes mechanistic molecular biology experiments on the molecular processing of *individual* lesions, as irradiated cells sustain DSBs in a stochastic manner at different numbers and severity that are randomly distributed throughout the genome. As a result, analysis of effects is only possible by theoretical modeling (39).

The above difficulties and shortcomings suggest that the field will benefit from molecular biology approaches allowing induction and processing analysis for specific forms of DSBs generated at specific locations in the genome. With such model-DSBs, the probability associated with each form to be processed correctly or incorrectly by each of the available repair pathways can be estimated. This information may subsequently be fed as a defined constant in mathematical models, reducing thus the number of free parameters and increasing the predictive power of the model. In the following sections, we describe such biological approaches and explain strengths and limitations.

## Endonuclease-Induced DSBs: The Simplest Form

**Figure 1:**
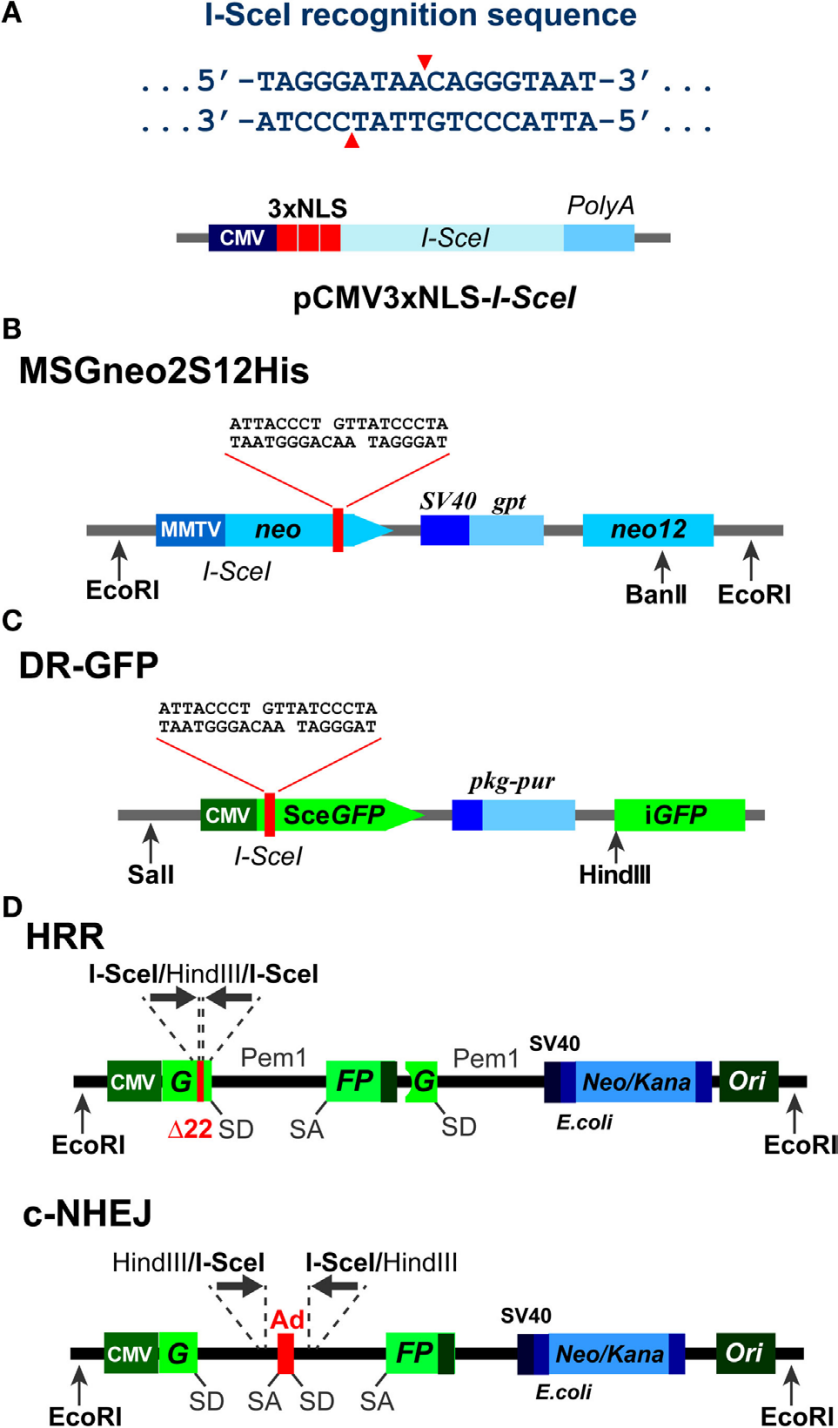
**Background information on I-*Sce*I based constructs**. **(A)** Top: recognition sequence of I-*Sce*I endonuclease. Note the generation of 3′ 4-bp overhangs. Bottom: domain structure of an expression vector, pCMV3xnls-I-*Sce*I, used frequently in transient transfection experiments to express I-*Sce*I (85). Note the three NLS sites that ensure the nuclear localization of the expressed enzyme. **(B)** Schematic representation of the MSGneo2S12His neomycin reporter vector developed to specifically analyze repair of the I-*Sce*I DSB by HRR (86). **(C)** Schematic representation of the DR-GFP vector developed to specifically analyze repair of the I-*Sce*I DSB by HRR (87). The GFP signal allows analysis by flow cytometry 1–3 days after transfection. **(D)** Schematic representation of reporter constructs utilizing the Pem1 intron and the Ad2 exon elements, and specifically developed to analyze repair of the I-*Sce*I-DSB by HRR and c-NHEJ, respectively (88).

Almost a decade ago, a fundamentally new approach for analyzing the effects of DSBs in living cells was introduced using rare cutting homing endonucleases. The most widely used member of this family of enzymes is the *Saccharomyces cerevisiae* I-*Sce*I endonuclease. As already mentioned, I-*Sce*I recognizes a unique 18-bp long DNA sequence (Figure 1A), which is absent from the human and mouse genomes. Thus, in order for I-*Sce*I to generate a DSB in these genomes, its recognition sequence must first be inserted using molecular biology approaches. Subsequent expression of I-*Sce*I will generate a DSB, specifically at the site of integration of the recognition sequence, which can be preselected or random (83, 84). Expression of I-*Sce*I must be transient and can be mediated by transient transfection of constitutively expressing vectors, or by the proper activation of an inducible enzyme.

In the most typical application of this model system, the I-*Sce*I recognition sequence is combined in a construct including a reporter gene [e.g., neomycin or in recent reporter assays, green fluorescence protein (GFP)] and located to interrupt its expression. Restoration of reporter expression serves as readout for the operation of a particular repair pathway in the processing of the DSB. These reporter constructs are in their majority integrated in the genome and are appropriately designed to evaluate repair efficiency through homologous recombination repair (HRR), classical non-homologous end joining (c-NHEJ), alternative end joining (alt-EJ), or single-strand annealing (SSA). Thus, analysis of DSB processing by a specific DSB repair pathway requires the construction of the appropriate vector and its integration into the genome.

In initial studies, I-*Sce*I was utilized to induce a DSB between two inactive *neomycin (neo)* direct repeat genes integrated into the genome of CHO cells, processing of which by homologous recombination generated a functional *neo* gene (86, 89) (Figure 1B). In these constructs, the fist *neo* allele is inactivated by the I-*Sce*I recognition sequence. The second *neo* allele is promoterless or truncated and may carry silent single-base substitutions that create restriction sites useful in product characterization through restriction fragment length polymorphism analysis. In the native state of this construct, *neo* is not expressed, and cells are sensitive to neomycin. However, after I-*Sce*I-mediated DSB induction, gene conversion may generate a functional *neo* gene and thus also neomycin-resistant clones. Such events are considered to reflect successful processing of the DSB by HRR.

More recently, DR-GFP reporter systems based on two directly repeated copies of the gene encoding GFP have been developed in the laboratory of Dr. Jasin (87) (Figure 1C) and find in different forms wide application in the field. In this system, gene conversion events result in expression of GFP, which can be quantitated by flow cytometry. The two mutated GFP genes are oriented as direct repeats and are separated by a puromycin *N*-acetyltransferase gene, which allows selection for cells carrying the construct. Distinct advantage of this version of the assay is that results are typically available 1–3 days after transfection of the I-*Sce*I expression plasmid. Analysis of neomycin resistance, on the other hand, requires 1–2 weeks.

**Figure 2:**
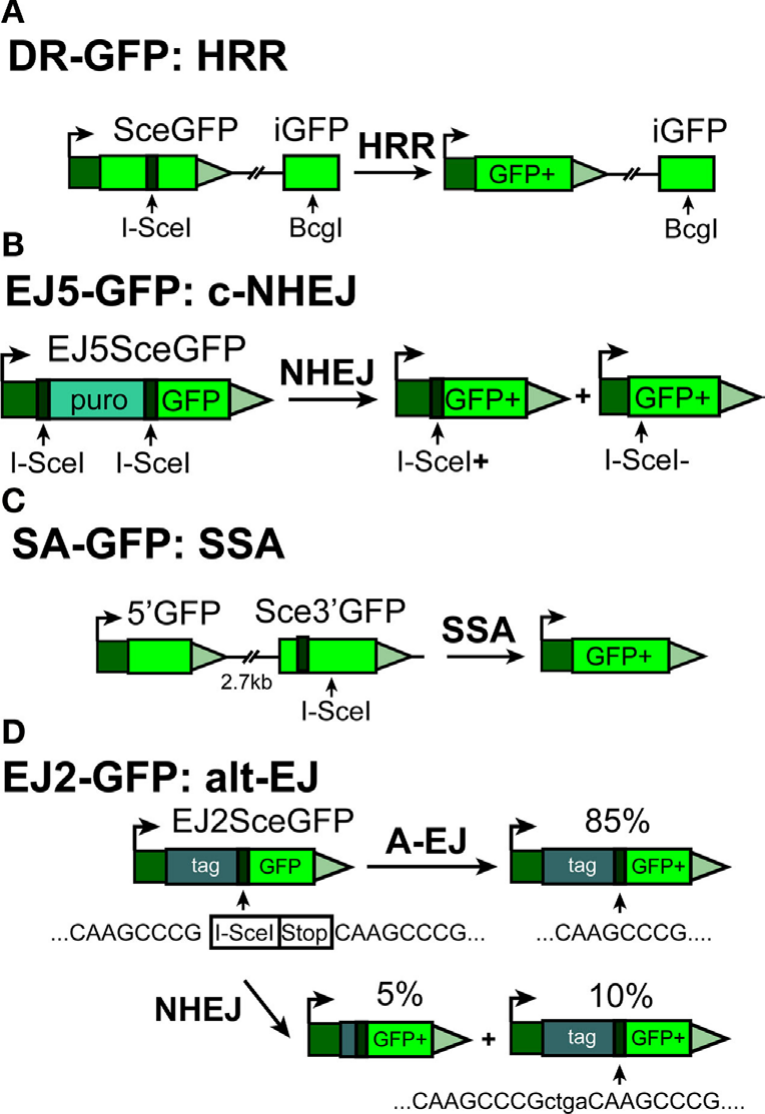
**Outline of reporter constructs developed in the laboratory of Dr. J. Stark (90) to analyze efficiency of I-*Sce*I-DSB-processing by different DSB repair pathways in human cells**. **(A)** DR-GFP construct for analyzing HRR. **(B)** EJ5-GFP for analyzing c-NHEJ **(C)** SA-GFP construct for analyzing single-strand annealing (SSA) and **(D)** EJ2-GFP construct for analyzing alt-EJ.

The DR-GFP reporter system has been successfully adapted to human cells, resulting in generation of U2OS–DR-GFP cells (90) (Figure 2A). Similar to the previously described system, one of the GFP genes in DR-GFP, SceGFP, is mutated by the insertion of the I-*Sce*I recognition site, while the second, internal GFP fragment, iGFP, located 821 bp downstream from SceGFP, has lost its active promotor element.

All reporter systems described above are designed to determine the activity of HRR in the processing of a single DSB induced by I-*Sce*I endonuclease. In addition to the above systems, a set of GFP-based fluorescent reporter constructs has been generated by Gorbunova et al. (88, 91) (Figure 1D), allowing analysis of NHEJ and HRR. These constructs are also based on artificially engineered GFP genes containing I-*Sce*I recognition sites for the induction of a DSB. In their native state, the integrated constructs do not express GFP as a result of an N-terminal truncation and mutations in the duplicated gene (in the case of the HRR construct), or by the integration within the GFP gene of an exon (Ad) flanked by the Pem1 intron elements (in case of the NHEJ construct). Here again, successful repair of the I-*Sce*I-induced DSB by NHEJ or HRR will restore the GFP gene, an event that is quantitated by flow cytometry.

Green fluorescence protein-based reporter substrates are now also available to specifically assess c-NHEJ, SSA, or alt-EJ (29, 90, 92, 93). Most of these constructs rely on the principles described above, but include elements allowing analysis of a specific DSB repair pathway (Figures 2A–D).

As already mentioned, use of I-*Sce*I as DSB inducer requires integration of its recognition sequence in the genome of human, mouse, or hamster cells. During the last few years, alternative approaches have been developed using endonucleases for which recognition sequences are present in the genome. One of these systems utilizes the I-*Ppo*I endonuclease, a member of a His–Cys box family of homing endonucleases isolated from the myxomycetous *Physarum polycephalum* (94), to induce multiple DSBs in the human genome (95–97).

**Figure 3:**
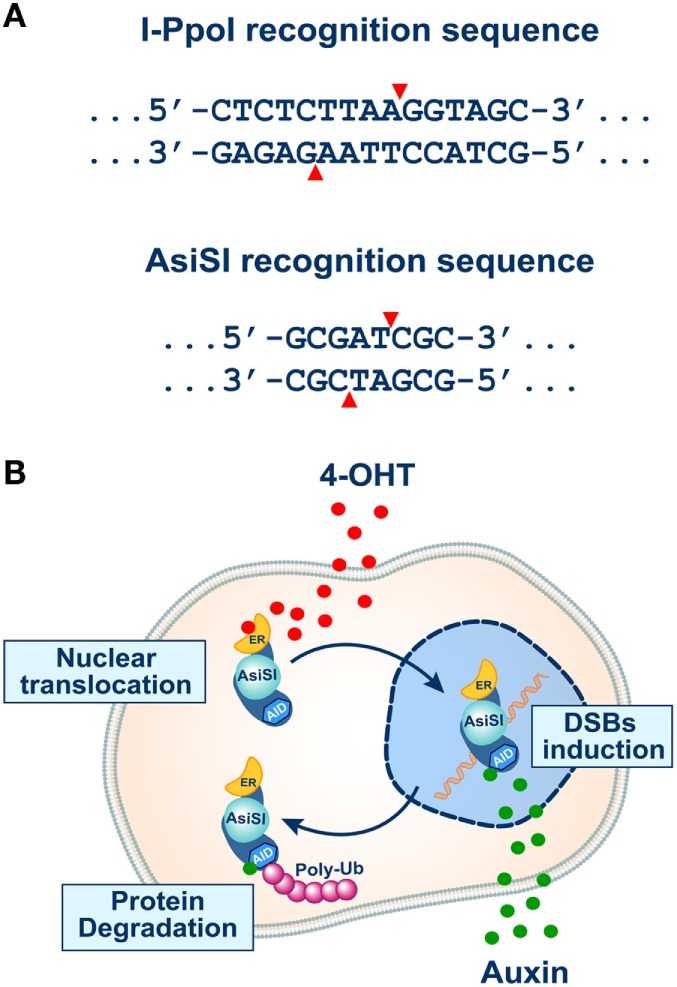
**(A)** Recognition sequences of I-*Ppo*I (top) and *Asi*SI (bottom) endonucleases (see text for details). Note that both endonucleases generate 3′ overhangs. **(B)** The inducible system of *Asi*SI endonuclease developed in the laboratory of Dr. Legube (102). The DIvA cell line expresses a form of the *Asi*SI endonuclease fused to estrogen receptor (ER) and the auxin-inducible degron (AID). The enzyme sequesters under normal conditions in the cytoplasm unable to reach the nucleus and thus to induce DSBs. Administration of tamoxifen (4-OHT) causes efficient translocation of the enzyme to the cell nucleus and the induction of DSBs (top part of the schematic). In this system, the endonuclease activity of *Asi*SI can be rapidly turned off by removing 4-OHT and administering auxin that activates the degron element and causes ubiquitin-mediated degradation of the enzyme (bottom part of the schematic).

I-*Ppo*I is a relatively small enzyme (18–20 kDa), operating as a homodimer, which in its natural host functions to cleave the highly conserved 15-bp ribosomal DNA homing sites (Figure 3A) to generate target intron transposition or “homing” (98). Expression of I-*Ppo*I in human cells causes cleavage of approximately 10% of the identified I-*Ppo*I genomic target sites (200–300 per genome) (95), generating 20–30 DSBs per cell, equivalent to the number of DSBs introduced by 1 Gy of X-rays. For increased versatility, a system has been developed using an I-*Ppo*I fused to a mutant estrogen receptor hormone-binding domain. The fusion protein stays constitutively in the cytoplasm unable to generate DSBs. Translocation into the nucleus can be mediated by incubation with 4-hydroxytamoxifen (4-OHT) (99–101), allowing thus the regulated induction of DSBs.

This system allows characterization of several features of the DSB response in human cells and has certain advantages over I-*Sce*I-based systems. First, I-*Ppo*I sites are present at well-known locations in the genome, which obviates their artificial introduction. Second, evolutionary conservation of the endogenous I-*Ppo*I sites permits DSBs to be introduced and assays to be performed in virtually any eukaryotic cell line. Third, as I-*Ppo*I induces multiple DSBs in the genome, full activation of the DNA damage response ensues, which allows analyses that go beyond repair pathway utilization.

An elegant assay along similar lines has also been proposed by Aymard et al. (103). These investigators developed a cellular system harboring a stable integration of a gene expressing the rare-cutting *Asi*SI restriction nuclease, which targets an 8-bp double-stranded DNA sequence (Figure 3A) and cleaves between the T and C to generate a 2-bases, 3′ overhanging ends (25, 104, 105). The genome-integrated *Asi*SI endonuclease in this model system is also fused to a modified estrogen-receptor ligand-binding domain. Thus, treatment of cells with 4-OHT triggers nuclear localization of the *Asi*SI enzyme and the rapid induction of approximately 150 sequence-specific DSBs dispersed across the genome (25, 104). This system provides a unique opportunity to simultaneously study, at a molecular level, repair events that transpire at many different DSBs located within various known chromatin locations (103).

Furthermore, as the *Asi*SI cleavage-sites are known, it is possible to use chromatin immunoprecipitation (ChIP) to directly monitor recruitment of repair factors onto damaged chromatin (103). Using this approach, it could be demonstrated that DSBs induced across the genome are not repaired by the same DSB repair pathway, and that transcriptionally active, H3K36me3 enriched, chromatin is preferentially repaired by homologous recombination, thereby pointing out a critical role of pre-existing chromatin state as determinant of DSB repair pathway selection (103).

As with the I-*Sce*I and I-*Ppo*I systems, the *Asi*SI system does not allow analysis of DSB repair kinetics because the enzyme remains present in the nucleus for prolonged periods of time and can cut repeatedly. To reduce this limitation, the same group added an auxin-inducible degron (AID) to *Asi*SI-ER fusion nuclease, thus allowing fast and efficient degradation upon auxin addition (102, 106) (Figure 3B). A similar improvement has also been successfully introduced in the I-*Sce*I system and was coupled with an extension allowing parallel analysis of HRR and alt-EJ (29).

## Zn-F Nucleases and TALENs: Tools for Site-Specific DSB Generation

**Figure 4:**
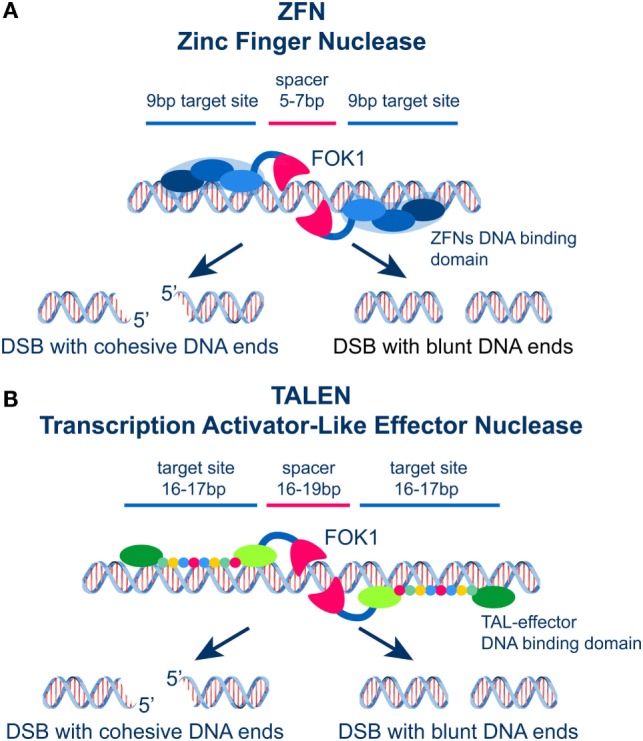
**(A)** Schematic representation of ZFNs showing the DNA binding and the FOK1 nuclease domains. Two zinc fingers bind left and right the site of DSB generation and localize the activity of FOK1 in a DNA molecule. The DNA binding domains are frequently designed to recognize a 9 bp target sequence. The FOK1 nuclease cuts the DNA strand 5–7 bp 3′ of the target site. Depending on the design of the target sites, expression of a ZNF nuclease will result in the formation of cohesive or blunt DNA ends. **(B)** Schematic representation of TALENs showing the DNA binding and the FOK1 nuclease domains. Targeting sites and spacer regions are indicated. Here again, cohesive or blunt ends are generated at the DSB depending on the selection of the recognition sites left and right the DSB.

Before the discovery of the CRISPR/Cas9 system that rapidly overtakes all previous systems (see below), significant effort and investment in resources was placed in two families of site-specific nucleases: the zinc-finger nucleases (ZFNs) and the transcription activator-like effector nucleases (TALENs). Both families of engineered proteins have a chimeric design with a common nuclease domain and a DNA-binding domain (Figure 4). In both families, DSB formation is mainly catalyzed by FOK1 endonuclease (107, 108) that generates, depending on the design, cohesive, or blunt DNA ends without sequence specificity (Figure 4). Yet, the concepts underlying the DNA-binding characteristics of ZFNs and TALENs are distinct and responsible for their inherent strengths and limitations. Their versatility arises from the ability to customize through molecular biology approaches their DNA-binding domains in ways that allow the recognition of virtually any DNA sequence.

The Cys2–His2 zinc-finger domain is among the most common types of DNA-binding motifs in eukaryotes operating in a widely different array of DNA sequences. It actually represents the second most frequently encoded protein domain in the human genome. The ZFN technology harnesses this biological evolution. An individual zinc-finger consists of approximately 30 amino acids in a conserved ββa (beta-sheet, beta-sheet, and alpha-helix) motif configuration. Each zinc-finger domain contacts 3 or 4 bp in the major groove of the DNA (108). In this technology, different zinc-finger motifs are combined to generate ZFNs that recognize the desired sequence 9 bp left and right from the target region (Figure 4A). The two components of the ZFN cut the corresponding DNA strands using FOK1, which is attached to the C-terminus of the ZFN modules (Figure 4A). Moreover, the cleavage domain requires the 5′ edge of each binding site to be separated by a 5–7 bp spacer region (Figure 4A).

Despite distinct strengths, the construction of ZFNs is complex requiring extensive know-how; it is very time consuming and shows limited flexibility in terms of engineering proteins recognizing any DNA sequence. As a result, their utilization, even before the advent of the CRIPSR/Cas9 system, had given way to the much more flexible TALENS.

TALENs contain TALE repeats of about 33–35 amino acids that recognize a *single* base pair *via* two hypervariable residues (repeat-variable di-residues, RVDs) (108). Combined TALE repeats can recognize a specific DNA target site of about 17 bp in length (Figure 4B). As a result of this unique property, TALENs can be easily and flexibly engineered to recognize DNA sequences of arbitrarily chosen lengths and compositions. For the application of TALENs discussed here, the number and location of the induced DSBs will depend on the frequency and the location in the genome of the selected sequence used to design the nuclease (109, 110). Thus, sequences can be selected and TALENs designed inducing in the genome a single DSB or multiple DSBs in variable configurations, depending on the specific question addressed.

The TALEN technology is powerful and flexible, and engineering of a site specific TALEN can be accelerated by “off-the-shelf” components that are combined to generate a functional protein within 1–2 weeks. Although the second generation of TALEN technology further improves on the distinct advantages of the approach, the work required to generate and test a single site specific TALEN nuclease is still considerable (111). As a result, this technology is also rapidly losing ground to CRIPR/Cas9 technology.

## The CRISPR/Cas9 System: Great Versatility, Short Learning Curve

**Figure 5:**
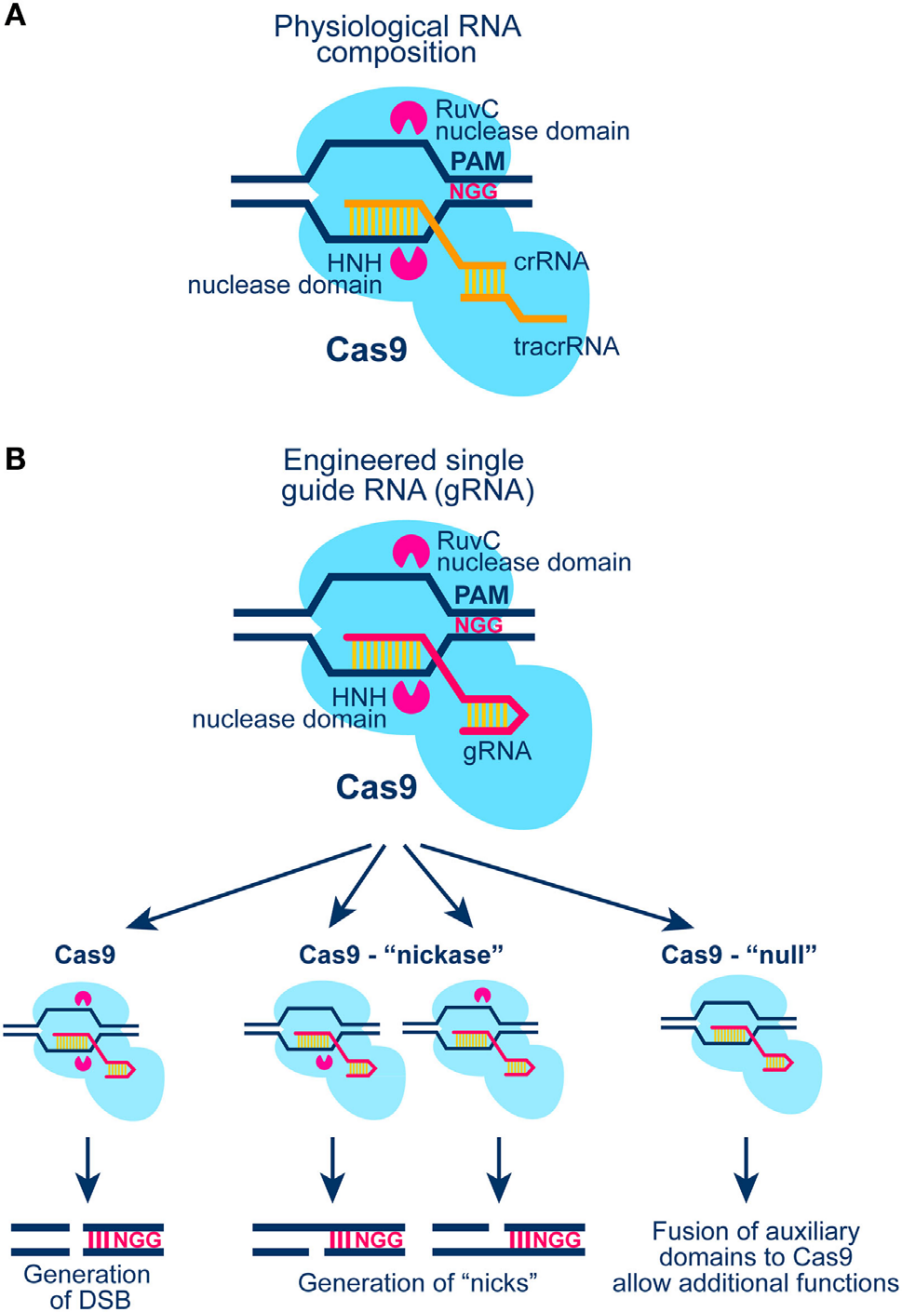
**(A)** Schematic representation of the CRISPR/Cas9 system in *S. Pyogenes* (see text for details). Cas9 generates blunt ends by cutting 3 bp downstream the PAM region of the target DNA molecule. **(B)** Schematic representation of Cas9 activation with a chimeric gRNA combining crRNA and tracrRNA (see A; see also text for more details). Cas9 nuclease harbors two nuclease domains: HNH and RuvC-like; mutation of one or both of these nuclease domains results in Cas9 enzymes with “nickase” properties, or with null nuclease activity. The latter form of Cas9 can be tethered to other proteins for gain-of-function DNA sequence-specific operations.

The CRISPR/Cas9 system with all its applications and potential is arguably the most rapidly expanding and evolving field in modern biology (Figure 5). From the initial discovery of the system as part of bacteria immunity to its modification for sequence specific genome editing, the technology has gone through a series of revolutionary developments that have been extensively reviewed (112–114). Relevant for the present outline is the potential of the system to generate a DSB anywhere in the genome by targeting at the specific location the Cas9 nuclease. Cas9 cuts the DNA as shown in Figure 5 to generate blunt DNA ends, guided by a partially complementary RNA molecule (gRNA). In its current stage of development, gRNA carries in addition to the sequence required for the proper targeting of Cas9 to the DNA molecule also a sequence for its activation.

Originally, Clustered Regularly Interspaced Short Palindromic Repeats (CRISPR) were found in the genome of *Escherichia coli*, but their function remained unknown until recently, when it was shown that these genetic elements are essential for the development of resistance against bacteriophages (115). Moreover, the CRISPR-associated protein 9 (Cas9) was described as a RNA-guided DNA endonuclease associated with the CRISPR-adaptive “immune” system in *Streptococcus pyogenes* (115, 116).

Three types of CRISPR systems have been identified thus far; from these forms type II is the most widely studied and the most relevant to the present outline. In type II CRISPR system, invading DNA is nucleolytically processed into small fragments (approximately 20 bp) that are incorporated into the CRISPR locus. This locus is transcribed, and transcripts are then processed to generate small CRISPR-RNAs (crRNA), which together with a trans-activating-CRISPR-RNA (tracrRNA) guide Cas9 to digest invading DNA upon repeat encounter (Figure 5A).

An important element of Cas9 activation is the Protospacer Adjacent Motif (PAM) at the target DNA sequence, which is essential for interactions between Cas9 and DNA (Figure 5A). Early work revealed that all three components (Cas9 protein, mature crRNA and tracrRNA) are required for efficient recruitment to and digestion of the target DNA sequence. A major development in the field was the recognition that crRNA and tracrRNA can be combined to generate a single guide RNA (gRNA) that enables all operations required for the targeted function of Cas9 (Figure 5B). This development greatly simplified the evolution of a large array of applications and forms the basis of the applications described here.

**Figure 6:**
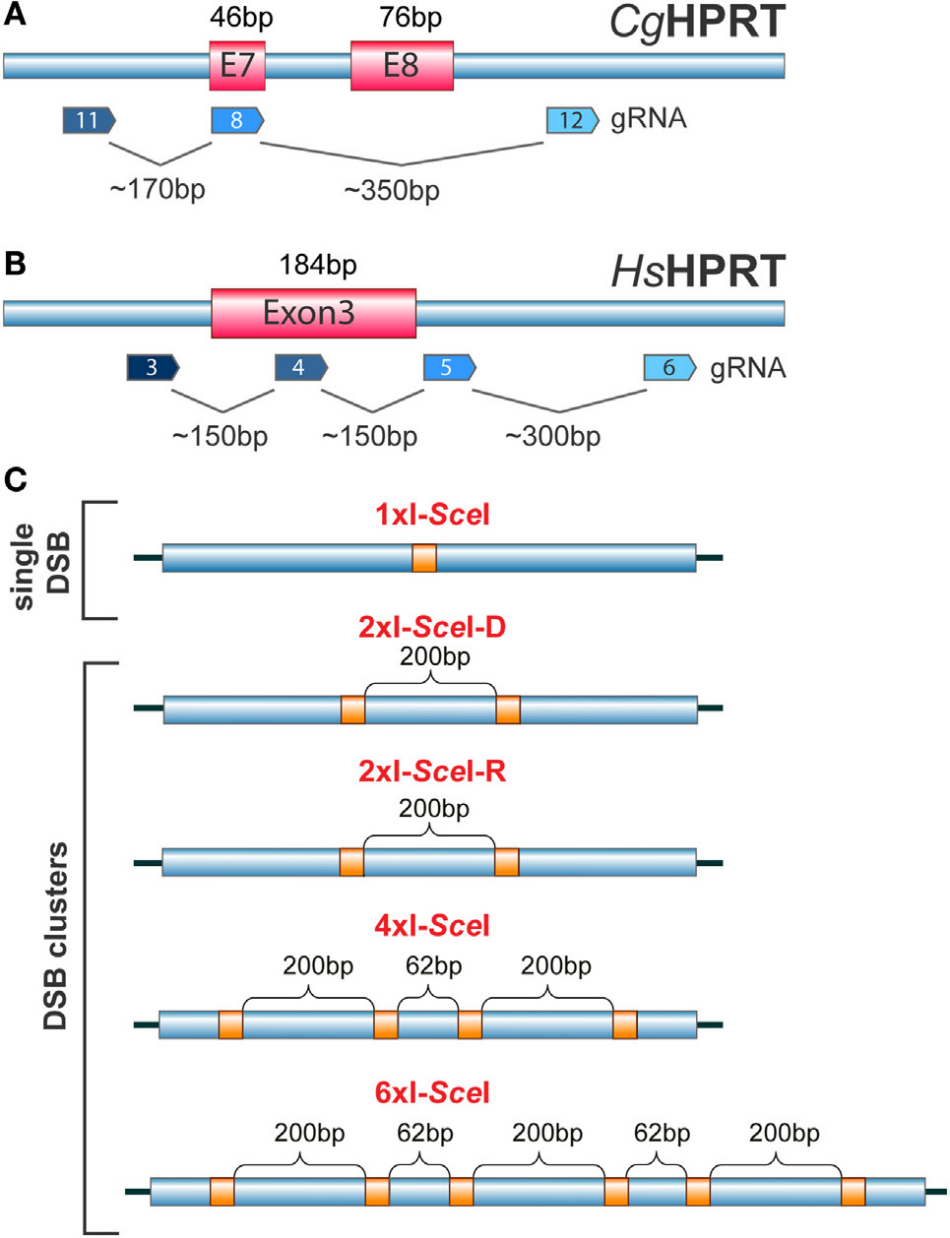
**(A)** Organization of the HPRT gene in the Chinese hamster *C. griseous* (*Cg*) near exons 7 and 8. Possible recognition sites of gRNAs allowing the generation of DSBs at different locations within exons and introns are indicated. **(B)** Exon 3 of the HPRT gene in *H. sapiens* (*Hs*). Possible recognition sites for gRNAs allowing the generation of single DSBs or DSB clusters within exons and introns are indicated. **(C)** Constructs carrying different combinations of I-*Sce*I sites engineered at different distances to model DSB clusters of increasing complexity. The schematic shows I-*Sce*I constructs that would generate upon integration in the genome of a cell, single DSBs, DSB pairs, DSB quadruplets or a cluster of six DSBs. The distances shown are arbitrary and chosen only for illustration purposes.

The number of applications utilizing CRISPR/Cas9-related genome editing/manipulation approaches is increasing exponentially with time. The technology is also very powerful for studies on the effects of single DSBs and DSB clusters in the mammalian genome (117, 118). Thus, existing CRISPR/Cas9 systems (116, 119) can be combined with appropriately designed gRNAs with the aim of inducing single DSBs or DSB clusters of different complexity within exons or introns of selected genes and study consequences in cell survival, genome integrity, DSB-response, or gene function. Figures 6A,B show as an example possible site selections for DSB induction within the HPRT gene, since it has been extensively used in the past to study IR-induced mutation induction (120, 121). Here, single DSBs and DSB clusters are induced at selected locations throughout the gene and at various constellations by combining different gRNAs (Figure 6).

The CRISPR/Cas9 technology is extremely powerful and promises to revolutionize the field by virtue of its ability to generate DSBs with great ease at any location of a known genome. In addition, mutated forms of Cas9 can be introduced in which one or both endonuclease domains are inactivated, thus generating enzymes with “nickase” or “null” activity (Figure 5B). Combination of Cas9 “nickase” with other systems of DSB generation, including the I-*Sce*I system described below, will allow testing of the biological consequences of a single-strand break in the vicinity of a DSB (complex DSB). Finally, fusions between a non-functional Cas9 enzyme and protein domains generating additional forms of DNA damage will further expand the spectrum of experiments investigating DSB complexity as a parameter in biological responses (Figure 5B). As with the I-*Sce*I, I-*Ppo*I, and the *Asi*SI systems, Cas9 will also generate repeated DSBs in the genome and its prolonged presence in the cell nucleus precludes analysis of DSB repair kinetics.

The fact that the generation of each DSB using the CRISPR/Cas9 technology requires individual gRNAs restricts somewhat its application for generating multiple DSBs, which at times may be a desirable outcome (see next session). This is because the generation of multiple single DSBs or DSB clusters at different genomic locations will require a number of gRNAs. This problem may be partly overcome by designing gRNAs, which recognize sequences in the genome that are repeated several times – excluding of course highly repeated DNA sequences. For example, many proteins contain common functional domains, encoded by similar if not identical DNA sequences, which could be targeted at once using a single gRNA molecule. An alternative solution for this limitation is offered by the model system described in the following section.

## I-*Sce*I-Based Models of DSB Clustering

We conclude this overview by outlining a recently introduced I-*Sce*I-based model system, complementary to the system outlined in the previous section using the CRISPR/Cas9 technology that allows direct analysis of assumptions regarding the biological effects of multiple single DSBs and DSB clusters (54). The model system utilizes transposon technology (122) to generate clonal cell lines with multiple genomic integrations of constructs carrying I-*Sce*I restriction sites at arrangements selected depending on the specific question addressed (Figure 6C). Cleavage of these sites by transient or conditional expression of I-*Sce*I to generate single DSBs or DSB clusters at different numbers (typically 1–15) and constellations allow analysis of the biological consequences at different endpoints. First results obtained using this model system (54) indicate that DSB clusters compromise c-NHEJ and possibly HRR, leaving alt-EJ as last resort in DSB processing.

Application of the same technology to mutant cell lines with defects in different aspects of DSB repair will allow extensive analysis of the DSB repair pathways handling this form of damage (4, 123, 124).

## Concluding Remarks

It is evident from the above outline that numerous novel technologies are available that promise to revive and revolutionize the ways we address fundamental questions of radiation damage and the associated radiation responses. These technologies allow the testing in well-defined systems of key hypotheses of mathematical models of radiation action, and the generation of data that may help to reduce their free variables. Such systems will be particularly useful in the analysis and characterization of the role of single DSBs and DSB cluster formation in the biological responses of high LET radiation.

The system utilizing the I-*Sce*I meganuclease to generate single DSBs and DSB clusters at random locations in the genome will extend the successful application this enzyme saw during the past 15 years to new questions relevant to DNA damage response. Zn-finger nucleases and TALENs will perhaps remain useful in addressing related questions at specific settings. Certainly, the most promising technology is the one utilizing the CRISPR/Cas9 system to introduce DSBs at pre-selected locations in the unmodified genome, with a pre-defined constellation.

One aspect with all systems of enzymatic DSB generation that needs to be considered in comparisons with the effects of IR concerns the specifics of DSB induction. Thus, IR by virtue of its well defined and typically short exposure times induces DSBs through non-recurring, distinct energy deposition events; DSBs induced in this way are subsequently processed and terminally removed from the genome. Nucleases, on the other hand, through their prolonged presence after expression or activation in the cell nucleus, will generate cycles in which initial DSB induction and subsequent processing will be followed by additional cutting and processing cycles, which will in principle continue until repeat processing mutates the nuclease recognition sequence, or until enzyme expression or activity subside. Since both “solutions” require a relatively long window of time, which is also likely to be different for each individual DSB, they generate a condition of chronic assault to the DNA generating “chronic” DSBs. Such chronic DSBs may induce responses with facets not present to those generated by the single events of IR, and which may engage distinct processing mechanisms that change the ultimate outcome. Additional problems may arise from off-target effects, variable on-target cutting frequencies, and the induction of a single form of DSB these systems allow.

Despite these inherent limitations, the approaches described above promise to enrich our knowledge of the biological responses to DSBs, to improve the focus on this form of DNA damage, and to enhance the power and utility of mathematical modeling by generating first principles that can be used as starting points. Last but not least, they are likely to strengthen interactions between experimental radiation biologists and mathematical modelers.

## Author Contributions

VM and EM wrote the manuscript and participated in preparation of the figures. GI generated the main outline of the article and wrote the manuscript.

## Conflict of Interest Statement

The authors declare that the research was conducted in the absence of any commercial or financial relationships that could be construed as a potential conflict of interest.
